# Cooperative Energy Harvesting-Adaptive MAC Protocol for WBANs

**DOI:** 10.3390/s150612635

**Published:** 2015-05-28

**Authors:** Volker Esteves, Angelos Antonopoulos, Elli Kartsakli, Manel Puig-Vidal, Pere Miribel-Català, Christos Verikoukis

**Affiliations:** 1 Department of Electronics, University of Barcelona (UB), Barcelona 08028, Spain; E-Mails: volker.estevesfuentes.2013@ieee.org (V.E.); manel.puig@ub.edu (M.P.-V.); pmiribel@el.ub.edu (P.M.-C.); cverikoukis01@ub.edu (C.V.); 2 Department of Signal Theory and Communications (TSC), Technical University of Catalonia (UPC—BarcelonaTECH), C./ Esteve Terradas 7, C4-202P, Castelldefels 08860, Spain; E-Mails: angelos.antonopoulos@tsc.upc.edu

**Keywords:** MAC protocol design, cooperative communications, energy harvesting, WBANs

## Abstract

In this paper, we introduce a cooperative medium access control (MAC) protocol, named cooperative energy harvesting (CEH)-MAC, that adapts its operation to the energy harvesting (EH) conditions in wireless body area networks (WBANs). In particular, the proposed protocol exploits the EH information in order to set an idle time that allows the relay nodes to charge their batteries and complete the cooperation phase successfully. Extensive simulations have shown that CEH-MAC significantly improves the network performance in terms of throughput, delay and energy efficiency compared to the cooperative operation of the baseline IEEE 802.15.6 standard.

## 1. Introduction

The median age of the world population is rapidly increasing. The 2013 United Nations report shows that 11.7% of the world population is older than 60 years, and this percentage is expected to rise up to 21.1% in 2050 [1]. Furthermore, about 75% of noncommunicable diseases (*i.e.*, heart disease, cancer, diabetes and chronic lung diseases), responsible for 68% of deaths in 2012, affect people over 60 years [2]. Hence, older age is typically accompanied by increased medical expenditures (up to 17.9% of the USA gross domestic product in 2012 [3]) and a heavy burden on the healthcare system.

Current advances in wireless and sensor technologies can facilitate the early detection and management of diseases, thus reducing hospitalization and treatment costs and improving the patients' quality of life [4]. In particular, wireless body area networks (WBANs) support the interconnection of sensors placed on or within the human body, enabling the monitoring of vital signals by sensor nodes, the performance of specific actions (e.g., automatic drug delivery) by actuator nodes and act as a bridge between the patient and the medical personnel.

Recently, the IEEE 802.15.6 [5] standard has been ratified, specifying the physical (PHY) and the medium access control (MAC) layers for short-range wireless communication between ultra-low-power consumption devices in WBANs. Typically, a star topology is adopted, where the sensor nodes are directly connected to a unique coordinating node (*i.e.*, the WBAN hub). However, the standard also supports two-hop communication between the nodes and the hub [6], in order to overcome the particular propagation characteristics in the human environment that usually involve high signal attenuation. In the same context, the works in [7,8] propose cooperative schemes for WBANs, achieving considerable performance enhancements in terms of reduced latency and packet loss and increased decoding probability of the transmitted information at the destination.

The efficient energy management constitutes another key issue in WBANs, since there are constraints on the size and, therefore, on the capacity of the batteries that power the sensor nodes. Furthermore, the recharging or replacement of the batteries is not a trivial task, especially for implantable devices. Hence, a lot of research effort has been placed on the design of energy-efficient MAC protocols for WBANs [9–17]. The main idea is to promote low duty cycles of operation, permitting WBAN nodes to remain in low-power sleep mode for prolonged periods of time, while supporting collision-free packet transmission to avoid unnecessary wasting of energy in collisions and retransmissions.

All of the above solutions aim to prolong the network lifetime by reducing energy consumption. However, the concept of energy harvesting (EH), based on collecting energy from the environment or the human body and converting it into electrical energy, constitutes a more drastic approach to the energy problem. On the other hand, even though EH is a promising solution that could lead to autonomous network operation, with the current EH technology, the harvested energy rate is usually lower than the transceiver consumption rate. Hence, in order to harness the potential of EH, energy-aware channel access mechanisms that adapt the network operation to the available energy level are essential at the MAC layer. However, there are currently very few works that focus on MAC protocols for EH-enabled WBANs [18–21]. In these works, only direct connections are assumed, thus neglecting cooperative scenarios, where the use of relays complicates the system design and requires new flexible MAC protocols for the network.

In this paper, we focus on a cooperative network, where a number of relays collaborate with the source to ensure the successful packet delivery at the destination. However, due to the error-prone channel of WBANs, multiple retransmissions are often required. As a result, the available energy at the relays may be depleted before the completion of the cooperation phase, and further transmission attempts by the source are required, leading to performance degradation. To that end, we propose cooperative EH MAC (CEH-MAC), an adaptive MAC scheme that introduces a charging period that enables the network relays to harvest the required amount of energy in order to successfully complete the cooperation phase. This time is dynamically selected by the WBAN hub, based on the available energy level of the nodes, and can be also calculated through a heuristic formulation. Through extensive simulations, we show that significant improvements can be achieved in terms of throughput, delay and energy efficiency.

Section 2 briefly reviews the energy-efficient MAC protocols for WBANs in the current literature. The system model is presented in Section 3, while Section 4 introduces the proposed MAC protocol and provides a comprehensive operational example. The simulation setup and the performance evaluation are discussed in Section 5, whereas Section 6 is devoted to the conclusions of our work.

## 2. Related Work

In [9], the authors introduce a low-delay traffic-adaptive MAC (LDTA-MAC) protocol for WBANs, where the guaranteed time slots are dynamically allocated according to the traffic load. In [10,11], the authors employ a superframe structure, containing a configurable contention-based access period and a contention-free period. Wake-up tables are established to coordinate the transmission schedule of the nodes, whereas a wake-up radio mechanism is employed for emergency situations. The concept of wake-up radio as a means to prolong the autonomous operation of energy-hungry sensors has also been adopted in [12]. The use of a statistical frame containing flexible scheduling information has been proposed in [13], to increase the sleep time and maintain low duty cycles in each beacon period. A hybrid access protocol is also proposed in [14], where an adaptive slot allocation based on the traffic load is employed in the contention-free phase. In [15], the duty cycles of the nodes are determined based on the criticality of the monitored data. In [16], a context-aware MAC protocol has been presented, adapting the access mechanism, the transmission time and the sampling rate of the nodes to the time-varying traffic and channel conditions, to achieve higher efficiency and reliability. Recently, in [17], an adaptive MAC protocol for WBANs, named the network longevity enhancement by energy-aware medium access control protocol (NLEEAP), has been proposed. By exploiting opportunistic cooperation, NLEEAP reduces the total energy consumption without introducing any additional devices in the network.

With regard to EH, there is hitherto only a limited number of research efforts in the literature. In [18], the nodes are assigned different priorities and access methods based on the criticality of their data and the type of EH source. In particular, nodes with high priority data employ a contention-free polling access scheme, whereas contention-based access is used for nodes with normal priority traffic. In [19], the authors show that conventional scheduling algorithms are not suitable for energy harvesting scenarios, and they propose a novel scheme that schedules tasks based on their time constraints and the available energy levels. An adaptive transmission policy that maximizes detection and correct transmission of data in WBANs with EH capabilities has been presented in [20]. The proposed scheme, formulated as a Markov decision process, exploits information on the energy level of the nodes, the data generation process and the battery recharge state to select the appropriate transmission mode for each state of the system. In [21], a resource allocation optimization scheme for WBANs is proposed, aiming to provide sustainability and QoS provisioning. Sustainability is achieved by adapting the data generation rate of the sensors, taking into account the available energy of each node acquired through EH, so as to guarantee uninterrupted network operation. As a second step, the transmission power and rate are optimized, in order to maximize the QoS in the data delivery. In spite of the novel insights that the aforementioned works bring to the design of EH-aware communication protocols, the cooperation aspect is usually not taken into account. In the following sections, we focus on a cooperative MAC protocol that exploits the EH context information to improve the performance of WBANs.

## 3. System Model

**Figure 1:**
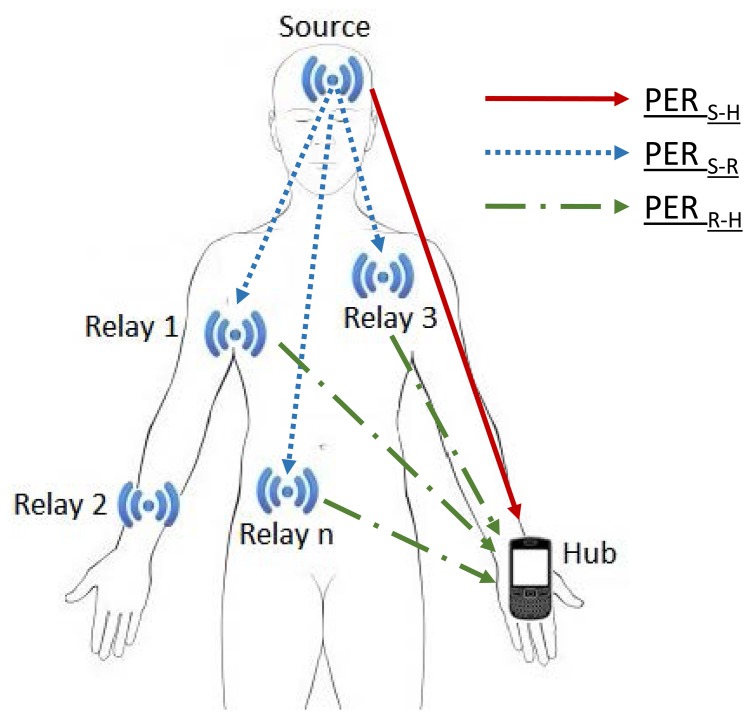
System model.

We consider a WBAN consisting of a source (*S*), a destination/hub (*H*) and *n* relay nodes (Figure 1). We assume that a direct link is established between the source and the hub, with a packet error rate (PER) denoted by *PER_S_*_−_*_H_*. However, whenever the direct transmission fails, the relays that have overheard the transmitted data enter a contention phase in order to attempt new retransmissions. Regarding the cooperative links, we assume a symmetric topology with identical channel errors in the links between the source and the relays (denoted by *PER_S_*_−_*_R_*) and in the links between the relays and the destination (denoted by *PER_R_*_−_*_H_*).

Regarding the power supply, we assume that all nodes (except for the hub) are powered by EH at a constant rate of *P_EH_*. On the other hand, the power consumption of the transceiver is denoted by *P_tx_*, *P_rx_* and *P_idle_* for the transmission, the reception and the idle mode, respectively.

## 4. Cooperative EH-Adaptive MAC Protocol

In this section, we present CEH-MAC, an IEEE 802.15.6-compatible MAC protocol that aims to improve the performance of cooperative WBANs by taking into account the available energy of the EH-powered nodes. The key idea of the proposed scheme is the introduction of a charging time (*T_charge_*) that enables the relay nodes to harvest sufficient energy for the completion of the cooperation phase. The parameter *T_charge_* is dynamically adjusted by the hub in each communication period, based on feedback information regarding the energy levels of the relays and the expected duration of the cooperation phase (depending on the number of relays, the channel conditions, *etc.*).

The remainder of this section is divided into three parts. First, the operation of a baseline cooperative IEEE 802.15.6-based scheme, denoted by Coop802.15.6, is described. Then, we present CEH-MAC, which enhances the baseline scheme, making it suitable for operation under EH conditions. Finally, an operational example of the proposed protocol is given.

### 4.1. Operation of the Baseline Scheme (Coop802.15.6)

We consider the following baseline cooperative scenario, compatible with the IEEE 802.15.6 standard for WBANs. In the beginning of each communication period, the source transmits a data packet to the hub. If the packet is received with errors due to the bad quality of the direct link (characterized by *PER_S_*_−_*_H_*), the hub initiates a cooperation phase by broadcasting a request for cooperation (RFC) packet. Then, the relays that have successfully received the original packet by the source enter a contention phase following the IEEE 802.15.6 carrier sense multiple access with collision avoidance (CSMA/CA) rules.

In CSMA/CA, a node has to sense the medium before transmitting. If the channel is idle during a short amount of time, the node executes a backoff mechanism by randomly selecting a counter within a specified contention window defined in the standard, which also depends on the user priority. The node keeps listening to the channel, and the backoff counter is decremented by one unit for each idle time slot. Channel access is gained when the counter reaches zero and the node attempts data transmission. The contention window size is doubled after a number of consecutive failures, and the process is repeated with a new backoff counter. Transmission failures may occur either due to packet collisions or due to channel errors (*PER_R_*_−_*_H_*). Hence, several retransmission attempts may be made before the correct reception by the hub, marked by the transmission of an acknowledgment (ACK) packet.

### 4.2. CEH-MAC Operation

The baseline scheme does not take into account the time-varying energy levels of EH-powered WBAN nodes, resulting in inefficient operation and performance degradation. More specifically, the energy consumed by the transceiver is usually higher than the energy collected through EH. As a result, when multiple retransmission attempts are required, the energy level of the relays is rapidly depleted, thus hindering the successful completion of the cooperation phase. In this case, the communication period is terminated without success, and consequently, the original packet should be retransmitted by the source, causing additional delays and eventual exhaustion of the source's energy.

CEH-MAC tackles this problem by introducing *T_charge_*, a dynamically-adjusted time period that takes place at the beginning of the cooperation phase. During this time, the relays remain idle, harvesting sufficient energy to complete the cooperation phase. The parameter *T_charge_* depends on the number of relays *n* and their energy levels, as well as the quality of the wireless link between the relays and the hub (*PER_R_*_−_*_h_*). The value of *T_charge_* is determined by the hub in each cooperation phase and is included in the RFC packet.

Four variables are required for the calculation of *T_charge_*:

(i) *E_est_*, which is the average energy level of the relays, estimated by the hub based on the received feedback. In particular, each relay includes its actual energy level, denoted by *E_act_*, in the retransmitted data packets. The hub extracts this information from the correctly received packets to calculate *E_est_*.
(ii) *E_req_*, which is the average required energy for the completion of the cooperation phase, considering all of the necessary retransmission attempts. Its value is calculated based on the average energy consumption in the previous cooperation phases.
(iii) *F_adj_*, which is an adjustment factor that accounts for the difference between the estimated *E_est_* and the actual energy level of the nodes (which is not known to the hub). The adjustment factor is generally a function of the number of relays *n* and the link quality *PER_R_*_−_*_H_* (*i.e.*, *F_adj_* = *f*(*n*, *PER_R_*_−_*_H_*)). The value of *F_adj_* increases after an incomplete (due to lack of energy) cooperation phase and decreases after a certain number of successful cooperation phases.
(iv) *P_gain_*, which is the actual power gain during charging, defined as the difference between the power harvested by the node (*P_EH_*) and the energy consumed under idle operation (*P_idle_*) (*i.e.*, *P_gain_* = *P_EH_* − *P_idle_*).

Based on these variables, *T_charge_* is calculated as:

(1) $$T_{\text{charge}}=\{\begin{array}{ll} \left(E_{\text{req}}\cdot F_{\text{adj}}-E_{\text{est}}\right)/P_{\text{gain}} & ,\text{if}\;E_{\text{req}}\cdot F_{\text{adj}}>E_{\text{est}}\\ 0 & ,\text{otherwise} \end{array}$$

**Figure 2:**
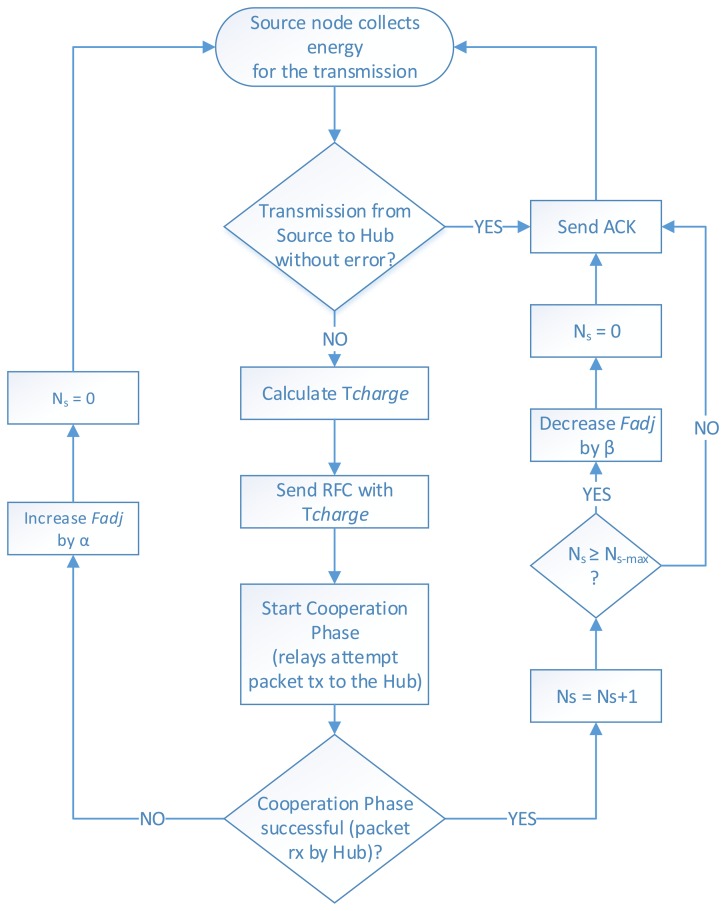
Cooperative energy harvesting (CEH)-MAC operation flowchart.

The operation of CEH-MAC is illustrated in Figure 2. When the source has a packet to transmit and a sufficient energy level, it attempts data transmission. If the hub receives the data successfully, it replies with an ACK, and the communication phase is completed. However, in the case that the packet is received with errors (and no ACK is issued), the source estimates the value of *T_charge_*, according to Equation (1), taking into account the estimated available energy at the relays, the required energy for the cooperation phase and the adjustment factor. Then, the source sends an RFC packet, including the *T_charge_* value, thus marking the beginning of the cooperation phase. During the cooperation phase, the relays attempt various retransmissions of the packet according to the IEEE 802.15.6 channel access rules, as described in Section 4.1. If the packet is correctly received by the hub, then an ACK is transmitted, and the cooperation phase is terminated with success. In this case, the counter *N_s_*, which denotes the number of consecutive successful cooperation phases, is also updated. When a maximum value equal to *N_s_*_−_*_max_* is reached (where *N_s_*_−_*_max_* is a system parameter), the *F_adj_* is decreased by a factor *β*. On the other hand, if the available energy of the relays becomes depleted before the successful reception of the data packet by the hub, the cooperation phase ends unsuccessfully. In that case, the *F_adj_* is increased by a factor *α*, thus ensuring a longer recharging time for the relays in the next round, and *N_s_* is reset to zero.

### 4.3. Operational Example

**Figure 3:**
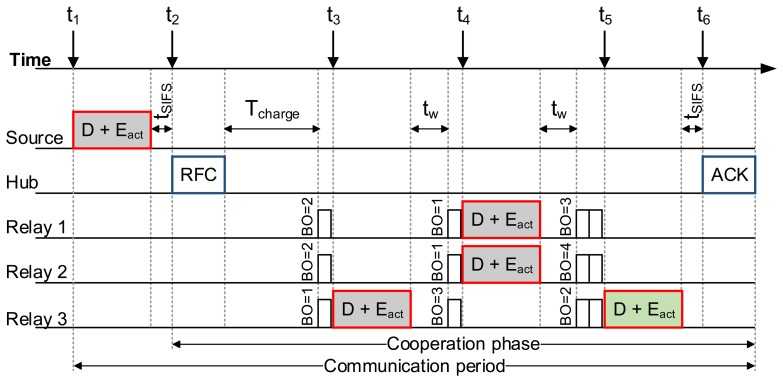
Operational example.

In continuation, we provide an example of the CEH-MAC operation (as depicted in Figure 3), considering *n* = 3 relays in the network. The key protocol steps are described next:

1. At t_1_, the source node transmits a data packet (D) to the hub, which also contains the available energy level *E_act_*.
2. At t_2_, the hub, upon receiving the packet with errors, calculates *T_charge_* and includes it in the RFC packet, which initiates the cooperation phase. Upon reception of the RFC packet, the source suspends its operation, whereas the relays remain idle to harvest energy for *T_charge_* time.
3. At t_3_, the relays that received the original data packet without errors participate in the cooperation phase, following the CSMA/CA protocol rules. In the example, Relay 3 gains access to the channel after one time slot and retransmits the packet D, including its *E_act_* value. However, the packet is received with errors by the hub. Hence, after a short period *t_w_* = *t_SIFS_*+*t_timeout_*, the relays resume the CSMA/CA contention.
4. At t_4_, Relays 1 and 2 simultaneously transmit a packet (again containing *E_act_*), leading to a collision. After *t_w_* elapses, CSMA/CA operation is resumed.
5. At t_5_, Relay 3 retransmits a packet (containing *E_act_*).
6. At *t*_6_, the hub, upon successfully receiving the data packet, transmits an ACK, concluding the cooperation phase. The hub also updates its variables. The value of E*_est_* is recalculated based on the *E_act_* level of Relay 3, included in the correctly received packet, while E*_req_* is updated according to the number of erroneous transmissions during the cooperation phase. Finally, F*_adj_* is modified, if necessary.

## 5. Performance Evaluation

We have implemented a system-level MATLAB simulator in order to assess the performance of the proposed protocol and to compare its performance with the baseline Coop802.15.6 scheme. In this section, we present the simulation scenario along with the adjustment factor (*F_adj_*) analysis and the simulation results.

### 5.1. Simulation Scenario

We consider the topology described in Figure 1, with one source node (S), one hub (H) and *n* relay nodes. Regarding the communication model, we consider saturated network conditions where the source node has always a packet to transmit in its buffer. In order to focus our study on the cooperation benefits, we assume that all direct transmissions contain errors, *i.e.*, *PER_S_*_−_*_H_* = 1, while the channel between the source and the relays is error free, *i.e.*, *PER_S_*_−_*_R_* = 0.

With regard to the energy parameters, all network nodes (apart from the hub) are powered by EH devices at a constant harvesting rate of *P_EH_* = 3 mW [22], while the power consumption of the transceivers has been selected according to [23]. Finally, the physical and the MAC layer parameters are compatible with the IEEE 802.15.6 standard [5]. Without loss of generality, we have considered the lowest priority specified in the standard for the relay nodes, in order to determine the limits of the contention window (*i.e.*, *CW_min_* and *CW_max_*). The simulation parameters are summarized in Table 1.

### 5.2. Adjustment Factor Analysis

**Figure 4:**
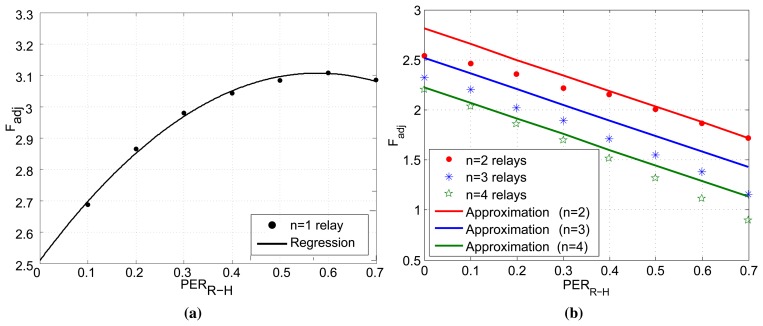
Adjustment factor (*F_adj_*) regression analysis. (**a**) *n* = 1; (**b**) *n* > 1.

Let us recall that the adjustment factor *F_adj_* is required for the calculation of the *T_charge_* value and can vary for different numbers of relays and packet error probabilities in the network. In this section, our goal is to analytically approximate this factor by applying the regression method in our simulation results. The results of this analysis are presented in Figure 4, where we can observe the different behavior of the factor for *n* = 1 (Figure 4a) and *n* > 1 (Figure 4b) relays in the network. In the case of *n* = 1, we are able to perform a regression analysis in a single set of data, and as a result, there is a very good match between the regression line and the experimental results. On the other hand, in the case of *n* > 1, we have followed a different approach, as the adjustment factor demonstrates a similar behavior. More specifically, instead of applying a single regression for every *n* (which would give more accurate lines, but different equations), we provide a common equation based on an adaptive regression that is able to sufficiently approximate the value of *F_adj_* as a function of the number of relays (*n*) and the channel conditions (*PER*). As a result, we have derived the following formula:

(2) $$F_{\text{adj}}=\{\begin{array}{ll} -1.8\cdot {\text{PER}}_{R-H}^{2}+2.1\cdot {\text{PER}}_{R-H}+2.5 & n=1\\ -0.3\cdot n-1.6\cdot {\text{PER}}_{R-H}+3.4 & n>1 \end{array}$$

It is also worth commenting on the origins of the different behaviors of *F_adj_*. This observation is very interesting, since, although counter-intuitive, it has a rational explanation. First, let us recall that this factor is an indication of the difference of the actual and the estimated energy in the nodes. Keeping this in mind, in the case of *n* = 1 relay in the network, this factor grows as the packet error rate increases, since there is only one relay in the network and the successful completion of the cooperation phase depends on the energy of this relay. As a result, as the PER increases, more retransmissions are required, and therefore, the relay needs more energy (*i.e.*, higher *F_adj_*) to carry out the cooperation phase. On the other hand, the presence of more than one relay in the network provides the system with diversity, as the retransmissions are shared among the relays. In addition, as the PER increases, the relays transmit more often, and the destination is able to perform a more accurate estimation about their energy, something that eventually implies lower *F_adj_*. Finally, in the same figure, we can also notice that the value of the factor also decreases as the number of relays in the network increases, as was expected.

### 5.3. Simulation Results

**Figure 5:**
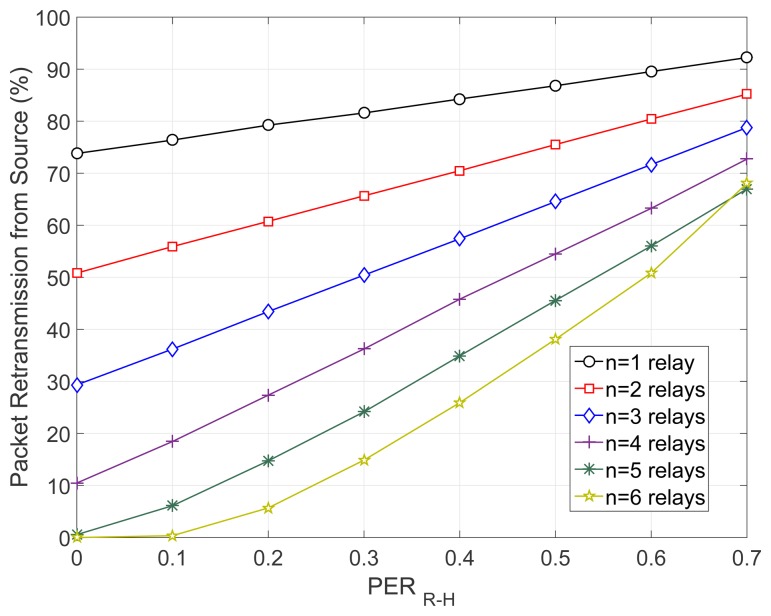
Percentage of retransmissions by the source in the baseline cooperative IEEE 802.15.6 (Coop802.15.6) scheme.

Initially, to highlight the necessity of our scheme, in Figure 5, we have plotted the percentage of the retransmissions that take place from the source in the baseline Coop802.15.6 scheme, where no EH awareness is considered. It should be mentioned that the source has to retransmit a packet only when the relay cooperation phase fails, which occurs when the energy of the relays is completely depleted. Hence, the percentage of source retransmissions is an indicator of the ineffective operation of Coop802.15.6, which has been a key motivator for the design of CEH-MAC. It can be observed that the percentage of retransmitted packets is generally very high, especially for a small number of relays. For instance, in the case of *n* = 1 relay, over 70% of the original data packets need to be retransmitted by the source node. As the number of relays grows, this percentage decreases, as the retransmissions are shared among the relays. However, in all cases, the retransmission percentage increases as the channel between the relays and the hub deteriorates (i.e., for higher values of *PER_R_*_−_*_H_*), and very high values are reached (e.g., exceeding 60% for *PER_R_*_−_*_H_* = 0.7 even when *n* = 6 relays are employed). Therefore, the baseline scheme seems to work properly only for specific topologies and channel conditions. For instance, considering *n* = 5 relays in the network and an error-free channel between the relays and the hub, a functional network can be achieved. However, such limited configurations can not realistically be applied in WBAN scenarios, where error-prone links are present and the number of relays is typically low, stressing the need for EH-aware MAC solutions.

**Figure 6:**
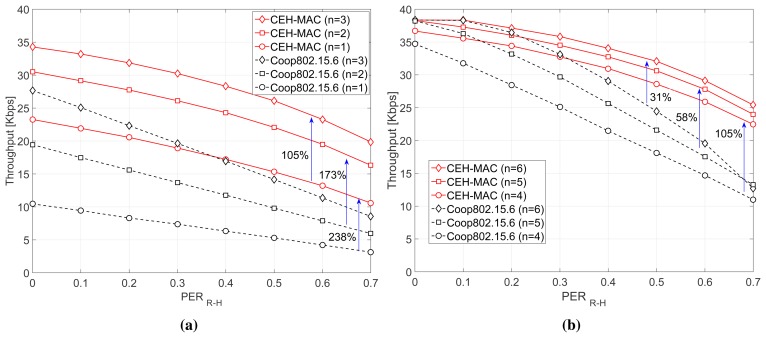
Throughput performance. (**a**) *n* = 1, 2, 3 relays; (**b**) *n* = 4, 5, 6 relays.

Figure 6 presents the throughput performance of the two schemes (CEH-MAC and Coop802.15.6) versus the *PER_R_*_−_*_H_*, for *n* = 1–3 relays (Figure 6a) and *n* = 4–6 relays (Figure 6b). Regardless of the number of relays, the proposed scheme outperforms the baseline operation, especially as the *PER_R_*_−_*_H_* value increases. The most remarkable performance gain is achieved for *n* = 1, where a throughput increase of 238% is achieved for *PER_R_*_−_*_H_* = 0.7. This demonstrates that, especially for a small number of relays (which is the most likely configuration in WBANs), the energy-aware policy adopted in CEH-MAC is crucial to guarantee the efficient operation of the network, whereas in the baseline scheme, the relay energy levels are rapidly depleted, leading to a low performance. Furthermore, it can be observed that throughput increases if more relays are incorporated in the network, since, in this case, less time is spent in the EH charging state (*i.e.*, the *T_charge_* takes smaller values). On the other hand, the throughput decreases as the *PER_R_*_−_*_H_* grows, since more retransmissions are needed, and consequently, the relays need longer time to store adequate energy, thus limiting the available time for data transmissions. Finally, it can be observed that, in some specific cases, the two protocols exhibit a similar performance, but this only occurs for a higher number of relays and under very good channel conditions (e.g., a similar throughput between CEH-MAC and the baseline is achieved for *n* ≥ 5 relays and *PER_R_*_−_*_H_* = 0), since both schemes exploit the high diversity of the multiple relays. However, as the channel condition deteriorates, CEH-MAC achieves a considerably better performance by allowing the relays to harvest sufficient energy in order to complete the cooperation phase.

**Figure 7:**
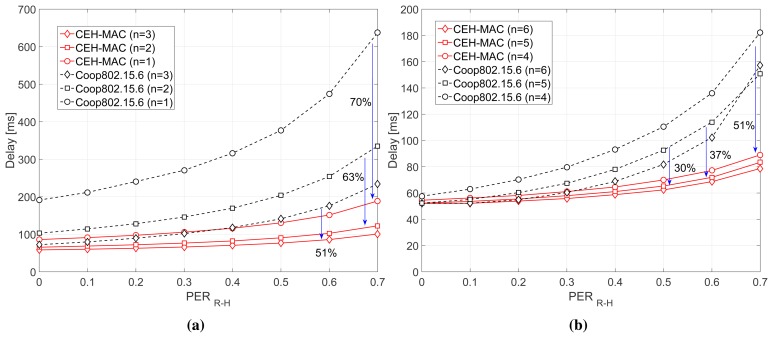
Delay performance. (**a**) *n* = 1, 2, 3 relays; (**b**) *n* = 4, 5, 6 relays.

Figure 7 presents the average end-to-end packet delay of the two schemes, again for *n* = 1−3 relays (Figure 7a) and *n* = 4−6 relays (Figure 7b). By applying the proposed scheme, we are able to decrease the packet delay up to 70% and 63% for the case of one and two relays, respectively. It is worth noticing that the packet delay in the baseline scheme is significantly increased for high *PER_R_*_−_*_H_* due to the incomplete cooperation phases. More specifically, in the case of highly erroneous channels, the relays waste all of their energy without success, and the source has to re-initiate the process. However, the application of CEH-MAC guarantees that the relays have sufficient energy to retransmit the packets, reducing the expected end-to-end packet delay in the network. In addition, in most cases, we can see that CEH-MAC is able to satisfy the delay constraint of 125 ms [24], which is particularly important in medical applications.

**Figure 8:**
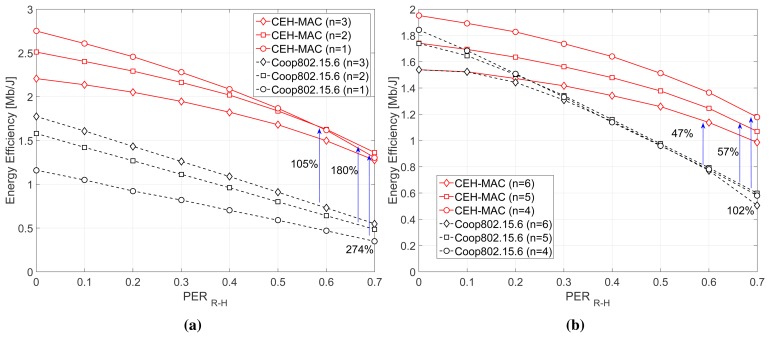
Energy efficiency performance. (**a**) *n* = 1, 2, 3 relays; (**b**) *n* = 4, 5, 6 relays.

Figure 8 shows the total energy efficiency in the network versus the *PER_R_*_−_*_H_*, again for *n* = 1−3 relays (Figure 8a) and *n* = 4−6 relays (Figure 8b). As we can observe, the significant enhancement in the network performance (i.e., throughput and delay) also has a direct impact on the energy efficiency, improving it up to 274% under certain network conditions (*i.e.*, *n* = 1 and *PER_R_*_−_*_H_* = 0.7). In this figure, we also notice an interesting tradeoff in the energy efficiency of the proposed scheme. In particular, higher energy efficiency is generally observed for a smaller number of relays, since high throughput can be achieved at a lower energy cost. However, as the *PER_R_*_−_*_H_* increases, the relative difference between the performance as a function of the relay number gradually decreases. For instance, the network achieves higher energy efficiency for *n* = 1 relay for low values of *PER_R_*_−_*_H_*; however, a better performance is achieved for *n* = 2 relays for *PER_R_*_−_*_H_* ≥ 0.6.

Finally, to assess the system's robustness, we have conducted simulation experiments considering a relatively high error probability in the link between the source and the relays, *i.e.*, *PER_S_*_−_*_R_* = 0.35. Table 2 presents the minimum (always for *PER_R_*_−_*_H_* = 0) and the maximum (always for *PER_R_*_−_*_H_* = 0.7) gains of the proposed CEH-MAC protocol compared to the baseline scenario with regard to the three metrics under study (throughput, delay, energy efficiency) in the case of one and two relays in the network. In the same table, we also list the minimum and maximum gains for the error-free case (*i.e.*, *PER_S_*_−_*_R_* = 0), as they can be found in Figures 6, Figures 7–8, to facilitate the comparison. As can be observed, although the increased PER in the first hop reduces the improvement gains of the CEH-MAC, the enhancement is still significant. The reduction can be explained by the fact that the errors between the source and the relays hinder the cooperation, regardless of the energy harvesting awareness, since the relays do not receive the packets correctly. Therefore, the performance of both schemes deteriorates, but we can still achieve important gains up to 202%, 66% and 230% for the throughput, the end-to-end delay and the network energy efficiency, respectively.

## 6. Conclusions

In this paper, we presented a cooperative MAC protocol, namely CEH-MAC, that exploits the EH information to improve the performance of WBANs. The proposed protocol adapts its operation to the EH conditions and enables the relay nodes to store sufficient energy in order to perform the retransmissions that are required to complete the cooperation phase. Extensive simulation experiments have shown that the proposed scheme significantly outperforms the baseline protocol (*i.e.*, without EH awareness), improving the network throughput, the average end-to-end delay and the total energy efficiency in the network. In our future work, we plan to analytically study the protocol's performance and incorporate advanced mechanisms (e.g., network coding) to further enhance its operation.

## Figures and Tables

**Table 1:** Simulation parameters. RFC, request for cooperation.

**Parameter**	**Value**	**Parameter**	**Value**
*P_EH_*	3 mW	Data packet	2112 bits
*P_tx_*	5.9 mW	RFC, ACK	136 bits
*P_rx_*	4.8 mW	[*CW_min_*, *CW_max_*]	[16,64]
*P_idle_*	1.7 mW	CSMA slot	145 μs
*α*	0.01	*t_SIFS_*	75 μs
*β*	0.001	*N_s_*_−_*_max_*	500

**Table 2:** Performance gains.

**Relays**		**n=1**		**n=2**	

		**min%**	**max%**	**min%**	**max%**
**Throughput**	*PER_S_*_−_*_R_* = 0.0	122	237	56	173
	*PER_S_*_−_*_R_* = 0.35	79	202	41	163

**Delay**	*PER_S_*_−_*_R_* = 0.0	55	70	36	63
	*PER_S_*_−_*_R_* = 0.35	44	66	29	62

**Energy Efficiency**	*PER_S_*_−_*_R_* = 0.0	137	274	58	180
	*PER_S_*_−_*_R_* = 0.35	86	230	43	164
